# Distinguishing Functional DNA Words; A Method for Measuring Clustering Levels

**DOI:** 10.1038/srep41543

**Published:** 2017-01-27

**Authors:** Hanieh Moghaddasi, Khosrow Khalifeh, Amir Hossein Darooneh

**Affiliations:** 1 University of Zanjan, Department of Physics, Zanjan, P.O.Box 45196-313, Iran; 2 University of Zanjan, Department of Biology, Zanjan, P.O.Box 45196-313, Iran

## Abstract

Functional DNA sub-sequences and genome elements are spatially clustered through the genome just as keywords in literary texts. Therefore, some of the methods for ranking words in texts can also be used to compare different DNA sub-sequences. In analogy with the literary texts, here we claim that the distribution of distances between the successive sub-sequences (words) is *q*-exponential which is the distribution function in non-extensive statistical mechanics. Thus the *q*-parameter can be used as a measure of words clustering levels. Here, we analyzed the distribution of distances between consecutive occurrences of 16 possible dinucleotides in human chromosomes to obtain their corresponding *q*-parameters. We found that CG as a biologically important two-letter word concerning its methylation, has the highest clustering level. This finding shows the predicting ability of the method in biology. We also proposed that chromosome 18 with the largest value of *q*-parameter for promoters of genes is more sensitive to dietary and lifestyle. We extended our study to compare the genome of some selected organisms and concluded that the clustering level of CGs increases in higher evolutionary organisms compared to lower ones.

DNA molecules as ordered strings of genetic codes including Adenine (A), Guanine (G), Cytosine (C) and Thymine (T) contain all information required for an organism to retain its life and produce next generation. Cytosines can be epigenomically modified to methylcytosines by de novo and maintenance DNA methyltransferases. C5-cytosine methylation occurs predominantly in the CpG dinucleotide context.

DNA sequences as one-dimensional arrays of four nucleotides (A, C, T and G) can be considered as texts so that they can be analyzed from a linguistic point of view to discover their different linguistic features. It is believed that there is a meaningful relation between linguistic interpretation of sub-sequences and their biological significances^1^,^2^. Here, the important matter is how to define the alphabets and words, for example nucleotides may be assumed as letters and sequences of *n* consecutive nucleuotides (*n*-tuples) as words. Some genome elements like exons, introns and others can also play the role of words.

There are two main approaches in the linguistic analysis of text. The first one considers a given text as a whole and attributes a quantitative measure to it. The Zipf’s, Heaps’ and Menzerath-Altmann’s laws are examples of such an approach. The second approach looks at the details of the text and finds the relation between parts of the text, namely words. The keyword detection methods fall into this category. DNA sequences have been analyzed by both approaches^3^,^4^,^5^,^6^. If we provide a list of distinct words in a text (or corpus) and sort the words according to their frequency from the most frequent word in the first rank to the least one in the last rank, according to Zipf, there is a power law relationship between frequencies and ranks^7^. Mantegna *et al*. studied Zipf’s law for coding and non-coding regions and found that non-coding regions behave more closely to natural languages than coding regions^3^. They extended these studies in ref. 4 and compared the statistical properties of coding and non-coding regions by means of statistical linguistics. They reported that the *n*-tuple Zipf’s plot of non-coding DNA has a power regression in a wide range of ranks; while, it is logarithmic for coding regions. Heaps’ law concerns about the number of distinct words in a given text. Clearly by proceeding through the text from the beginning, the number of it’s distinct words grows. Therefore a power law relation exists between the size of the text part and the number of distinct words therein^8^. It was shown that the Heaps’ law should be modified for coding regions of DNA. It was also understood that DNA has a revealed difference with random sequences^9^. A kind of self-similarity is discovered in texts according to Menzerath-Altmann’s law^10^ which states that the average size of the text’s components tends to decrease as the number of the components increases. It is meaningful in different levels of texts including syllabus-letter, word-syllabus and sentence-word^9^. For instance, in word-syllabus level it means that the longer a word, the shorter the syllabuses. In the context of genome, this law in chromosome-based genome can be interpreted as species with more chromosomes tend to have less average chromosome size^10^. In ref. 11 this law was applied in gene-exon-based level and it has been shown that the number of exons in a gene increases as the mean size of exons decreases.

In literary texts, keywords are more correlated and clustered than common words^12^. Similarly, functional DNA sub-sequences form clusters. Some genome elements like genes, Transcription Factor Binding Sites (TFBSs) and CpG islands are shown to be clustered through the genome and are not randomly distributed^13^,^14^,^15^. Hence, the clustering level can be regarded as a measure to rank the biological sequence-based words according to their importance on the context of DNA. Accordingly, finding an appropriate algorithm is critical for detecting clustering of DNA words. Hackenberg *et al*. used fluctuations of distances between occurrences of each word to calculate the clustering level of each word on the DNA sequences of chromosomes. They observed a correlation between DNA keyword clustering and the enrichment of the corresponding words in functional genome elements^16^. In order to predict biological significant words, an algorithm was also introduced in ref. 17 to detect clusters of DNA words using their distance distributions; however the expected values by the method do not satisfactorily comply with the observed data. Provata *et al*. used fractal methods for comparing fractal dimensions of coding/non-coding regions of higher and lower eukaryotes^18^. Najafi and Darooneh showed that this finding may be interpreted as a method for ranking words in a text. They detected that the pattern of a word type locations in a text is a fractal; therefore, introduced degree of fractality as an index to rank the importance and complexity of words^19^. Statistical mechanics also appears as an appropriate tool in linguistic studies^20^. Non-extensive statistical mechanics as a generalization of Boltzman Gibbs (BG) statistical mechanics is successfully used to describe a wide range of complex systems such as natural and social systems^21^. The Tsallis entropy is the key concept in non-extensive statistical mechanics which can explain several features of complex systems and the distribution function is *q*-exponential which is a generalization of the ordinary exponential^20^. So *q*-parameter as the nonextensivity parameter shows the nonextensivity level of the system and has been applied effectively in various fields of study such as physics, chemistry, biology, economy and linguistics^22^. In this study we proposed and applied a method for finding the important sub-sequences in DNA. This method was firstly introduced by Mehri and Darooneh to extract the important words in a text using non-extensive statistical mechanics^22^. It is noticeable that in random distribution of words, the distribution function of distances between successive appearance of a word is exponential, while in the meaningful texts the words form clusters and their distribution functions are no longer exponential but *q*-exponential. Hence, the *q*-exponential as a generalization of the ordinary exponential is a suitable candidate for describing the clustering of words. In current work, we find the *q* parameter for all dinucleotides in different chromosomes of humans and predict their potential susceptibility for epigenetic modifications. As we find here, CG appears to be more clustered in genome elements of all organisms. Therefore it can be used for classification of organisms from evolutionary perspective.

## Methods

### Distribution of sub-sequences

The occurrence probability of a specific nucleotide (letter) in a DNA sequence is


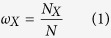


where *X* indicates one of the nucleotides (A, C, T and G). *N*_*X*_ is the total number of such nucleotide in the sequence and *N* is the length of the sequence (the total number of nucleotides) in terms of base pairs (*bp*). A given sub-sequence (word) has the generic form as *S: X*_1_*X*_2_…*X*_*L*_ where *X*_*i*_ is the nucleotide (letter) at the *i*-th position and *L* is the word length. In the pure random sequence, there is no correlation between occurrences of letters and they are completely independent. Therefore. the occurrence probability of word *S* is just the multiplication of it’s letters probabilities.


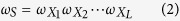


The probability of finding two consecutive occurrences of a given word at the distance *d* in terms of *bp* is,


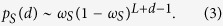


By imposing the normalization condition, we find,


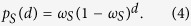


Equation 4 is the geometric distribution function for a discrete random variable. The geometric distribution is the only memory less discrete distribution. In practice, it is convenient to use the cumulative distribution function (cdf) which is defined by,


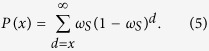


By using the geometric series formula, we obtain,


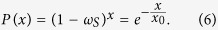


Where *x*_0_ = 1/|ln(1 − *ω*_*S*_)| and has a large value because *ω*_*S*_ ≪ 1. Thus, for purely random sequences, the cdf of the inter-word distances has exponential form.

As a matter of fact, the nucleotides are not randomly distributed and their occurrence probabilities are dependent on each other which means the presence of correlations and forming the clusters. For the clustered sequences, larger inter-word distances exist as well as smaller ones, i.e. existence of larger distances is more probable comparing to the random sequences. Therefore the cdf of inter-word distances is no longer exponential, but has a power law relationship for large distances. The *q*-exponential is a generalization of the ordinary exponential function using a real parameter *q* and exhibits a power law behavior in its tail and recovers the exponential function as a limiting case. This *q*-exponential function is defined as,


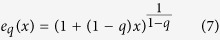


The parameter *q* is assumed to be a positive number. When it is close to one, *e*_*q*_(*x*) approaches to exp(*x*). For q > 1 and 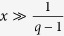 we have,


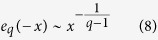


The inverse of the *q*-exponential is *q*-logarithmic function which is defined as,


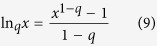


It is also a generalization of the logarithmic function and for *q* = 1 is just ln x.

We assume here that the cumulative distribution function of distances between consecutive occurrences of a word in a DNA sequence can be described by:


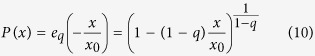


Equation 10 represents the Tsallis distribution which is arising from maximization of the Tsallis entropy under appropriate constraints in the context of the non-extensive statistical mechanics^20^. Nowadays non-extensive statistical mechanics appears as a powerful tool for studying systems with long range interactions between its components and/or systems with limited sizes and/or non-equilibrium ones^23^,^24^,^25^. Non-extensive statistical mechanics has been used previously to describe features of several systems with the aforementioned properties from quite various fields (examples can be found in Tsallis book)^21^. Texts with long range correlations between its components are also successfully analyzed via non-extensive statistical mechanics approach^22^. Similarly, biological systems ranging from genes and proteins to cells and organisms lie in the above-mentioned category of systems because of the long range correlations between some DNA words^26^. Thus, we assumed that non-extensive statistical mechanics would be a proper candidate to study these systems.

**Figure 1:**
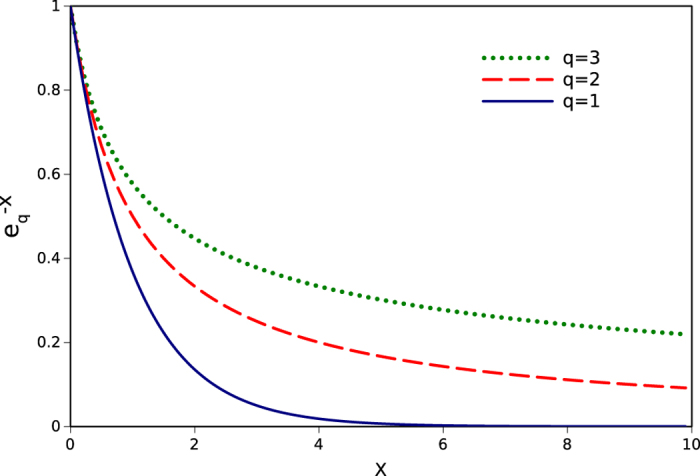
*q*-exponential function for three different values of *q*, ordinary exponential function or *q* = 1 (solid line), *q* = 2 (dashed line) and *q* = 3 (dotted line).

In Fig. 1 the *q*-exponential function, *e*_*q*_(−*x*), is plotted for different values of *q*. It is obvious that larger distances are more probable for larger *q* values. Therefore more deviation of *q* from one is equivalent to existence of larger distances which corresponds to the higher clustering quality. So the *q*-parameter can be considered as a quantitative measure of the clustering levels of different sequences. The clustering level is consequence of correlations in a sequence. Shuffling the sequence would surely destroy any correlations and drastically decrease the clustering quality of words with large *q*-value. The words with *q* ~ 1 are randomly distributed through the sequence and shuffling operation doesn’t change their *q*-values. Therefore we can assume that the clustering level demonstrates the importance of a word^19^.

### Ranking DNA words

Functional DNA sequences and genome elements are clustered through DNA sequences just as keywords in texts^12^,^13^,^14^. Therefore some of the methods for extracting keywords in texts might also be valid for DNA sequences. We use the method originated from non-extensive statistical mechanics (based on the value of *q*-parameter) to rank importance of different DNA sequences and genome elements^22^. In this method, the value of *q*-parameter associated with the cdf of inter-word distances,reveals the importance level of a word. This is because that larger *q* value means more clustering of word and more deviation from random distributions, as discussed earlier.

To apply this method, the first step is defining DNA words. Initially, we choose dinucleotides as DNA words. Considering their positions (coordinates) on all human chromosomes from UCSC database (www.genome.ucsc.edu), we obtain the distances between consecutive occurrences of each dinucleotide and calculate the frequencies of each distance. If a sub-sequence (word) occurs quite consecutively without any distances and/or occurs considering overlaps, all these sub-sequences are accounted for the original word. The cumulative distribution function of distances versus the distances is plotted for each dinucleotide to obtain its corresponding *q*-parameter. Then different dinucleotides can be compared based on the value of their *q*-parameters. This approach is also performed for other DNA words as trinucleotides and exons.

## Results

### Dinucleotides

**Figure 2:**
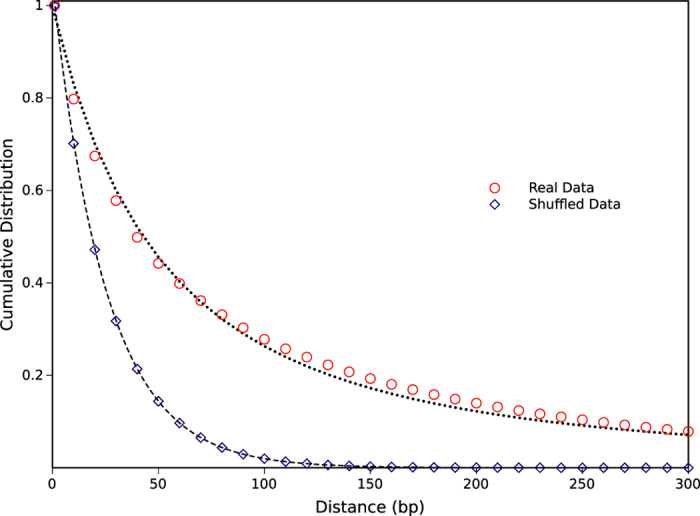
Cumulative distribution functions of distances between consecutive occurrences of CG dinucleotides on chromosome 1. The real and shuffled data are marked by circle and diamond, respectively. The dashed line is the fitting result for the shuffled data by exponential function. The dotted line shows the *q*-exponential fitting result for the real data.

Regarding four nucleotides (A, G, C and T) as letters, sixteen possible dinucleotides are assumed as different two-letter words. For each word, distances between consecutive occurrences are obtained and then the cumulative distribution functions of distances versus distances in terms of base pairs (bp) are plotted for all human chromosomes. It is found that the graphs for all dinucleotides on all human chromosomes are suitably fitted by *q*-exponential distribution function (Equation 8). The largest *R*^2^ and the smallest RMS (0.9999 and 0.0004, respectively) are obtained for TA dinucleotide on chrX; while, the smallest *R*^2^ and the largest RMS (0.9909 and 0.0134, respectively) are calculated for CG dinucleotide on chr4 indicating reasonable quality of fitting. It is shown in Fig. 2 for CG dinucleotide on chromosome 1 as an example. Similarly, the cumulative distribution function of distances is shown for the randomly shuffled data, where the whole sequences of chromosome 1 have been shuffled uniformly without changing the total number of each nucleotides. The difference between two graphs is thoroughly evident in Fig. 2. The distribution function for the shuffled sequences is fitted suitably by exponential function; while the real data is fitted quite appropriately by *q*-exponential function. Note that some data points of Fig. 2 are omitted. However all data points are used for fitting by *q*-exponential distribution function.

It’s worth mentioning that clustering on the whole chromosome implies the clustering level on average but due to various spatial variation across the genome, it is more convenient to discuss about the clustering level on specific regions essential for gene expression pattern. Hence, we have obtained q-parameter on the upstream regions of genes on all human chromosomes separately as well as on the whole chromosomes (Table 1). Dinucleotides with different *q*-parameters for some randomly chosen chromosomes are also shown in Table 1 as well as trinucleotides in Table 2 which indicates the importance levels of these sub-sequences.

The complete list of the obtained *q*-parameter for different dinucleotides on all chromosomes is available in Supplementary Material A and on upstream regions of genes in Supplementary Material B.

**Figure 3:**
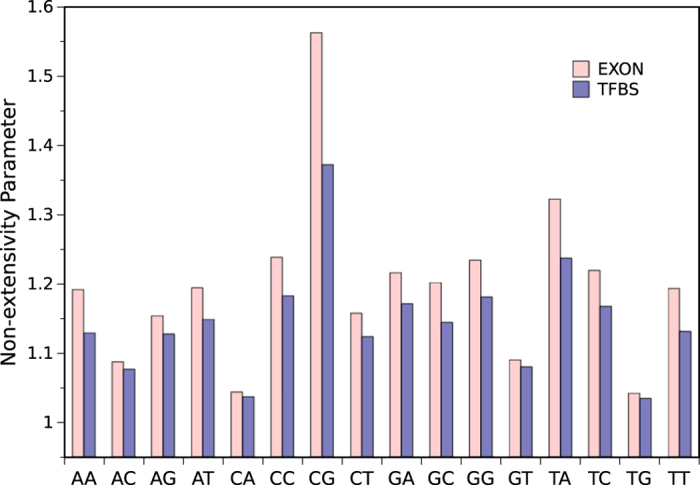
Comparison of dinucleotides *q*-parameters in exons and TFBSs.

We have also obtained *q*-parameters of dinucleotides in exons as well as TFBSs (Transcription Factor Binding Sites). As it is obvious in Fig. 3, *q*-parameters of all sixteen dinucleotides are larger in exons rather than TFBSs.

**Figure 4:**
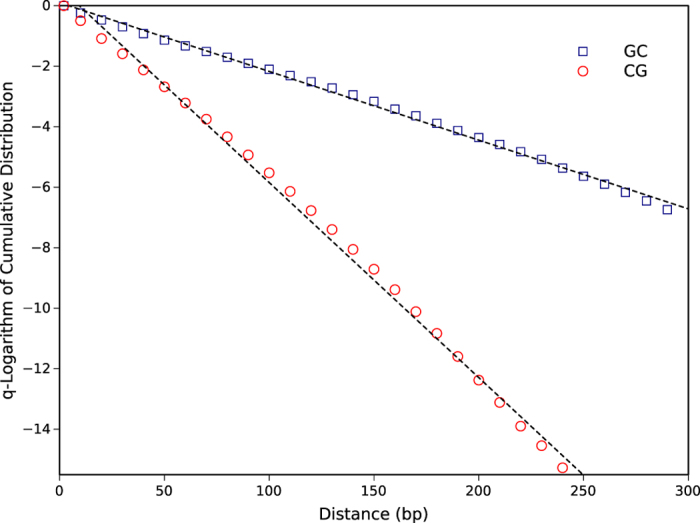
*q*-log of the cumulative distribution function of distances between consecutive occurrences of CG (circles) and GC (squares) dinucleotides versus distances for chromosome 21 as an instance. The linearity confirms the goodness of fits.

The *q*-log of the distances for CG and GC dinucleotides on chromosome 21 is shown in Fig. 4 as an example. Their linearity confirms the suitable fitting by Tsallis distribution function. The graph has steeper slope for CG in comparison to GC, this corresponds to having a larger *q*-parameter and therefore a higher clustering level which means more deviation from random distribution.

### Genome elements

**Figure 5:**
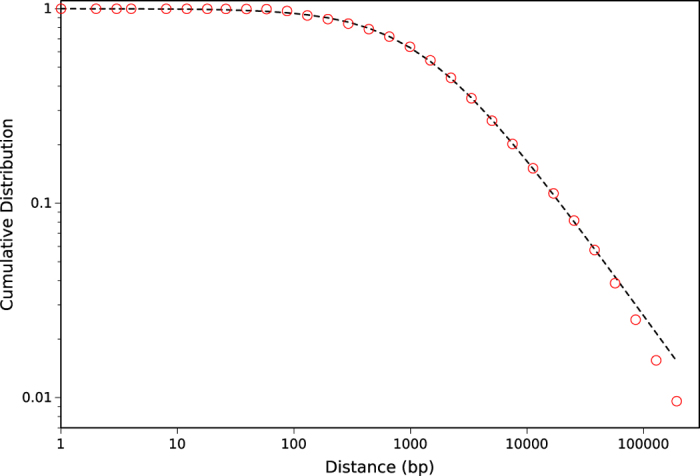
Cumulative distribution function of distances between exons on whole genome. Exons as DNA words with variable lengths also fit well with *q*-exponential function. Dashed line shows the fitting result.

To show that the applicability of this method is not restricted to words with the same lengths, we also applied the aforementioned method for analyzing genome elements of various sizes such as exons. For this purpose, we selected the exons of all human genome by considering positions of exons on each chromosome and obtained the distances between consecutive occurrences of exons. Figure 5 shows the cumulative distribution function of distances. The result is appropriately fitted by Tsallis distribution function. Note that some data points are omitted for better visibility of the graph, although all data points are used for fitting.

### Comparing different species

**Figure 6:**
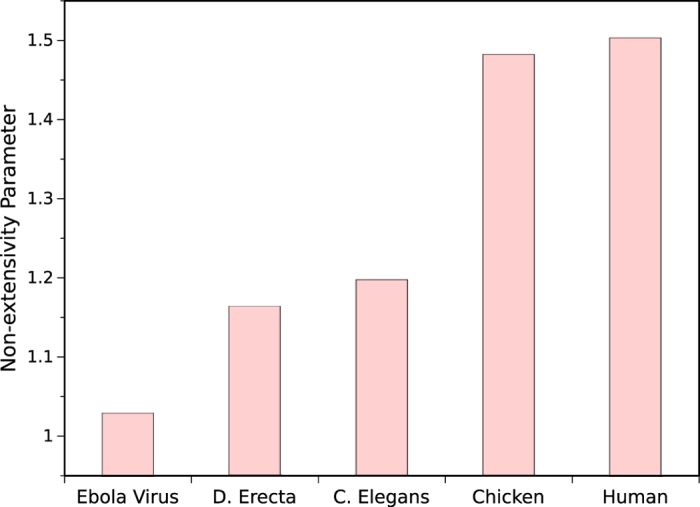
*q*-parameter of CG dinucleotide over whole genome for some selected organisms. Clustering level of CGs increases in higher evolutionary organisms compared to lower ones.

We extended our study by comparing the clustering levels of CG dinucleotide in the whole genome of some selected organisms including Ebola virus, D. erecta, C Elegance, chicken and human. The results of this comparison are provided in Fig. 6.

## Discussion

In order to quantify the clustering level of DNA-based words with a reasonable criterion, we have introduced well known q-parameter in statistical mechanics as a measure of ranking the importance of some defined DNA sequences and genome elements in human genome and the genome of different organisms.We have applied our method to compare different dinucleotides and found that CG dinucleotide has the largest *q*-parameter among the others not only on the whole chromosomes but also on the upstream regions of genes which reveals their higher clustering level compared to other dinucleotides in all human chromosomes (Supplementary Material A and B). The biological interpretation of this data is associated with the biological functions of CG element and its effect on the expression of genes as a consequence of epigenetic modification. This finding implies that CG is the most important two-letter word among the others; sixteen possible dinucleotides in the context of DNA sequences in all of the human chromosomes. Regarding CG clustering on the whole chromosome, it can be seen that chromosome 3 has the largest value of *q*-parameter indicating its higher tendency for spontaneous mutation to thymine during evolutionary time scale. It is also found that chromosome 18 has the largest value of *q*-parameter for upstream regions (Promoters) of genes when compared to other human chromosomes. Moreover, the chromosomes 8 and 10 have the lowest values of *q*-parameters. Therefore, it appears that the effect of chromosome 18 on differentiation of somatic cells during the developmental stage may be significant. It may also be more prone to epigenetic modification originated from environmental conditions such as dietary and lifestyle. As shown in Fig. 2, DNA words in a random distribution behave exponentially; while in the real case, where the words are correlated, their distribution function goes to *q*-exponential. Therefore, the amount of difference between these two cases for a specific word indicates it’s level of significance. In other words, the more deviation from the exponential function to *q*-exponential one reveals higher biological significance. Exons as the precursors of proteins are considered as one of the genetically important elements in cells. Resulting data of analyzing the clustering level of exons are in good agreement with the biological importance of exons as the coding regions of proteins. These findings together confirmed the ability of our methods in analyzing the genome sequences based on the clustering level of their elements and can be used for further analysis of genome sequences. Furthermore, comparisons of *q*-parameters for CG content at the genome of different organisms (Fig. 6) shows that the clustering level of CG dinucleotides in different organisms changes in a regular pattern in accord with the evolutionary time scale. More clustering of CG dinucleotide in more evolved and complex organisms can be attributed to gradual increase of specific functional and structural roles of CG dinucleotide inside DNA during evolutionary time. Since, the higher organisms such as human have genomes in which the *q*-parameter for CG dinucleotides is greater than that of evolutionary lower organisms and it may be concluded that higher organisms are more susceptible to environmental conditions in cases that these conditions can affect the phenotype of organisms based on the epigenetic modifications. This observation is in agreement with ref. 27, in which the authors analyzed the distribution of dinucleotides inside human and other organisms using distance-based approach and found a significant statistical distribution for CG of human genome; while for the other organisms studied they found more heterogeneity.

## Conclusion

In summary, regarding our method of analysis and biological interpretation of resulting data, it can be said that non-extensive entropy model is able to quantitatively distinguish the biologically important DNA words in the context of genomic sequence and provide a systematic insight to the sequence-based information of biological events at the level of genome. Considering the mass accumulation of protein sequences in bioinformatics databases, it appears that similar analysis for different protein families can provide valuable information appropriate for designing new variant of protein for biotechnological applications.

## Additional Information

**How to cite this article:** Moghaddasi, H. *et al*. Distinguishing Functional DNA Words; A Method for Measuring Clustering Levels. *Sci. Rep.* **7**, 41543; doi: 10.1038/srep41543 (2017).

**Publisher's note:** Springer Nature remains neutral with regard to jurisdictional claims in published maps and institutional affiliations.

## Supplementary Material

Supplementary Material A

Supplementary Material B

## Figures and Tables

**Table 1:** *q*-parameters for different dinucleotides on some randomly chosen human chromosomes in (a) on the upstream regions of genes and (b) on the whole chromosome.

Word	chr3a	chr3b	chr7a	chr7b	chr14a	chr14b	chr18a	chr18b	chr21a	chr21b
CG	1.8971	1.8537	1.8118	1.6237	1.7202	1.6253	2.4934	1.534	1.5883	1.6165
TA	1.2975	1.1545	1.3703	1.1784	1.3302	1.1696	1.2598	1.1419	1.3159	1.1897
GG	1.2194	1.2118	1.2651	1.2357	1.2470	1.2313	1.3284	1.2354	1.2565	1.2654
AC	1.1123	1.0248	1.1366	1.0244	1.1143	1.0249	1.0884	1.0228	1.0264	1.023
CA	1.0636	1.0169	1.0579	1.0166	1.0425	1.0178	1.0300	1.0167	1.0641	1.0191

**Table 2:** *q*-parameters for different trinucleotides on some randomly chosen human chromosomes.

Word	chr4	chr8	chr10	chr15	chr20
GCG	1.735146	1.675188	1.635335	1.609606	1.598388
CCG	1.726111	1.663821	1.633669	1.629512	1.644872
CGC	1.725597	1.665496	1.633301	1.613182	1.606054
CGG	1.719771	1.651277	1.605608	1.600651	1.578919
GAT	1.042450	1.037433	1.022208	1.021420	1.034390
ACT	1.027384	1.004085	1.002648	1.002119	1.002287
